# A Case Report of Rash at Peritoneal Dialysis Exit Site

**DOI:** 10.1177/2324709615618222

**Published:** 2015-11-27

**Authors:** Elvira O. Gosmanova, Ikena Ezumba, Kristopher R. Fisher, Kerry O. Cleveland

**Affiliations:** 1 Department of Medicine, University of Tennessee Health Science Center, Memphis, TN, USA; 2 Department of Dermatology, University of Tennessee Health Science Center, Memphis, TN, USA

**Keywords:** peritoneal dialysis catheter exit site, allergic contact dermatitis, gentamicin

## Abstract

The International Society for Peritoneal Dialysis recommends the regular application of topical antibiotic-containing preparations in addition to a routine exit site care to reduce the risk of exit site infection (ESI). Among these prophylactic antimicrobial preparations, topical gentamicin is one of the widely used and effective antibiotics for prevention of ESI and peritonitis in peritoneal dialysis (PD) patients. Overall, topical gentamicin is well tolerated; however, its use can be associated with the development of allergic contact dermatitis (ACD). We describe a first reported case of PD catheter exit site contact ACD due to topical gentamicin mimicking ESI. The patient in this report developed worsening violaceous in color and pruritic rash surrounding the PD catheter exit site that appeared 3 weeks after the initiation of gentamicin cream. The association between development of rash and initiation of topical gentamicin led to a suspicion of local reaction to gentamicin rather than ESI. Skin biopsy confirmed ACD. Discontinuation of the provoking agent and subsequent treatment with topical hydrocortisone application led to a resolution of the exit site rash. Any rash at a PD catheter exit site should be considered infectious until proven otherwise. However, it is important to be aware of noninfectious etiologies of exit site rashes as the treatment of these 2 conditions differs.

## Background

The development of rash at the peritoneal dialysis (PD) exit site is never trivial for nephrologists and is always concerning for PD exit site infection (ESI). ESI occurs in up to 20% of PD patients and is associated with a 6-fold increase in incidence of PD-associated peritonitis in the subsequent 60 days even with appropriate treatment of ESI.^1^ In turn, PD-associated peritonitis poses risks of PD catheter removal, recurrent hospitalizations, and even death.^2^ Therefore, the International Society for Peritoneal Dialysis (ISPD) recommends using antibiotic-containing preparations, in addition to a routine daily exit site care, to reduce incidence of ESI.^3^ However, it is important to be aware that antimicrobial preparations do not fully eliminate risk of ESI and, moreover, can be associated with noninfectious complications. We describe for the first time a clinical presentation and diagnostic approach to gentamicin-induced contact dermatitis at the PD catheter exit site that mimicked ESI.

## Clinical Presentation

**Figure 1.**
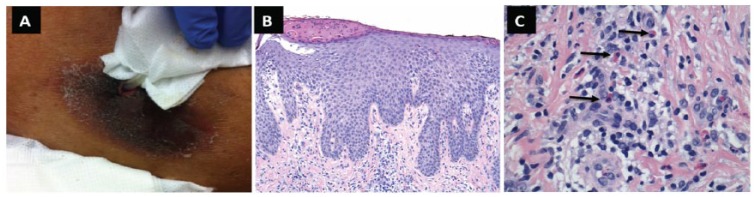
Clinical and histological presentation of gentamycin-induced contact dermatitis. (A) Presenting rash at the peritoneal dialysis catheter exit site. (B) Skin biopsy of the peritoneal dialysis exit site lesion showing spongiotic dermatitis (hematoxylin–eosin, 40×). (C) A higher magnification showing eosinophilic spongiosis; arrows point to eosinophils (hematoxylin–eosin, 200×).

A 54-year-old African American female was undergoing continuous cycling PD for end-stage renal disease due to diabetes. She presented to clinic with 2.5-week history of a worsening pruritic rash around her PD catheter exit site. Three weeks earlier she began topical gentamicin sulfate 0.1% cream for ESI prophylaxis. The patient reported no fever or abdominal pain. On examination, she had normal vital signs. The PD catheter exit site had an 8.5 × 4.5 cm ovoid crusted plaque, violaceous in color with a peripheral rim of erythema and without granulation (Figure 1A). There was no tenderness, swelling, or drainage present at the PD catheter exit site. Abdomen was nontender with normoactive bowel sounds and peritoneal fluid was clear.

Clinical diagnosis of allergic contact dermatitis (ACD) due to gentamicin was made and gentamicin cream was discontinued. Skin biopsy performed the following day demonstrated psoriasiform spongiotic dermatitis with eosinophils, consistent with ACD (Figure 1B and C). The exit site lesion slowly resolved with residual mild hyperpigmentation after stopping gentamicin cream and initiating hydrocortisone 2.5% cream. Mupirocin 2% cream was subsequently added for ESI prophylaxis with no recurrence of exit site rash.

## Final Diagnosis

ACD due to gentamicin cream

## Discussion

ESI should be considered with the development of rash at the PD catheter exit site. ESI is a major risk factor for the development of PD-associated peritonitis; therefore, prompt diagnosis and treatment of ESI are essential.^3-5^ The most common pathogens causing ESI are *Staphylococcus aureus* and *Pseudomonas aeruginosa*. Additional pathogens leading to ESI include *Staphylococcus epidermidis, Escherichia coli*, diptheroids, streptococci, nontuberculous mycobacteria, and fungi.^3,4^ The ISPD recommends cleaning of PD catheter exit site with antiseptic agent and application of topical antimicrobials, such as gentamicin or mupirocin, for the prevention of ESI.^3^ Topical gentamicin has been shown to reduce ESI and peritonitis due to gram-positive and gram-negative organisms;^6,7^ while, topical mupirocin mainly reduced ESI due to gram-positive organisms.^6,8^ ESI is typically diagnosed clinically based on the finding of purulent or bloody drainage from PD catheter exit site, surrounding erythema, tenderness, and swelling. However, the presence of skin rash and erythema without drainage at the PD catheter exit site can be also due to early infection, allergic reaction to PD catheter material,^9^ or to mechanical trauma.^4^ Additionally, allergic reactions to PD catheter exit site care products such as antibiotic preparations (mupirocin and polysporin)^10^ and antiseptic agents^11,12^ can manifest as skin rash around PD catheter exit site.

Erythema and rash around the exit site can be mistaken for ESI; however, absence of drainage, tenderness, and swelling may be clues for contact dermatitis. The diagnosis of contact dermatitis is usually established on clinical grounds based on characteristic appearance of rash, negative Gram stain and culture of exit site, and favorable response to withdrawal of suspected agent along with supportive measures such as topical steroid preparations. Skin biopsy can be used to confirm the diagnosis, and in our patient it demonstrated psoriasiform spongiotic dermatitis with eosinophils (Figure 1B and C).

Topical gentamicin (cream, ointment, eye and ear drops) has been previously linked to periocular ACD.^13^ In a randomized controlled trial comparing effectiveness of topical gentamicin sulfate 0.1% cream and mupirocin 2% cream, only minor exit site irritations developed in 10.5% of patients in both treatment arms with no reported cases requiring discontinuation of antimicrobial preparations.^6^ ACD is a cell-mediated type 4 delayed hypersensitivity reaction.^14^ ACD is observed more frequency in patients with atopic eczema, nickel sensitization, stasis dermatitis, and chronic actinic dermatitis.^15^ Patients who develop ACD from gentamicin are at an increased risk for generalized eczematous eruption following parenteral administration of gentamicin.^16^ Contact dermatitis from gentamicin can also result in cross-sensitivity to other aminoglycosides like neomycin.^17^ Of note, allergy to topical neomycin—the most commonly used topical antibiotic in the United States^18^—has been reported to occur in up to 13.1% of the general population.^19^

## Conclusions

ACD due to topical gentamicin is not uncommon and can occur at the PD catheter exit site. It is important to be aware of this association to avoid incorrect diagnosis of PD catheter ESI and inappropriately continued antibiotic use in cases of ACD. In contrast, the routine exit site care, including topical antimicrobials, is continued during PD catheter ESI. The failure to discontinue provoking allergens in ACD can lead to the worsening of exit site rash, incorrect diagnosis of refractory ESI infection, and potentially result in PD catheter removal.
